# Global Diversity of Aloricate Oligotrichea (Protista, Ciliophora, Spirotricha) in Marine and Brackish Sea Water

**DOI:** 10.1371/journal.pone.0022466

**Published:** 2011-08-10

**Authors:** Sabine Agatha

**Affiliations:** Department of Organismic Biology, University of Salzburg, Salzburg, Austria; University of Western Ontario, Canada

## Abstract

Oligotrichids and choreotrichids are ciliate taxa contributing to the multi-step microbial food web and episodically dominating the marine microzooplankton. The global diversity and distribution of aloricate Oligotrichea are unknown. Here, the geographic ranges of the 141 accepted species and their synonyms in marine and brackish sea water are analyzed, using hundreds of taxonomical and ecological studies; the quality of the records is simultaneously evaluated. The aloricate Oligotrichea match the moderate endemicity model, i.e., the majority (94) of morphospecies has a wide, occasionally cosmopolitan distribution, while 47 morphospecies show biogeographic patterns: they are restricted to single geographic regions and probably include 12 endemic morphospecies. These endemics are found in the Antarctic, North Pacific, and Black Sea, whereas the “flagship” species *Strombidinopsis cercionis* is confined to the Caribbean Sea. Concerning genera, again several geographic patterns are recognizable. The species richness is distinctly lower in the southern hemisphere than in the northern, ranging from nine morphospecies in the South Pacific to 95 in the North Atlantic; however, this pattern is probably caused by undersampling. Since the loss of species might affect higher trophical levels substantially, the aloricate Oligotrichea should not any longer be ignored in conservation issues. The ecophysiological diversity is considerably larger than the morphological, and even tops the richness of SSrRNA and ITS haplotypes, indicating that probably more than 83–89% of the diversity in aloricate Oligotrichea are unknown. The huge challenge to discover all these species can only be managed by combining the expertises of morphological taxonomists, molecular biologists, ecologists, and physiologists.

## Introduction

### History of Discovery

Based on morphologic and ontogenetic features, the Oligotrichea Bütschli, 1887 unite the halteriids, oligotrichids, and choreotrichids (see ‘Classification and phylogeny’ and Table 1 for scientific and vernacular names). While the former two taxa contain exclusively aloricate (naked) species, the choreotrichids embrace naked species and the loricate (house-forming) tintinnids, which are not considered in the present compilation.

**Table 1:** Classification of halteriid, oligotrichid, and aloricate choreotrichid ciliates; vernacular names are in bold; the numbers of marine and brackish water species are in brackets.

Spirotricha Bütschli, 1887** = spirotrichs**
Class Oligotrichea Bütschli, 1887 (141 aloricate species)
Subclass Halteriia Petz & Foissner, 1992** = halteriids** (2 species)
Order Halteriida Petz & Foissner, 1992 (2 species)
Family Halteriidae Claparède & Lachmann, 1859 (1 species)
Genus *Halteria* Dujardin, 1841 (0 species)
Genus *Pelagohalteria* Foissner, Skogstad & Pratt, 1988 (1 species)
Family Meseridae Corliss, 1961 (1 species)
Genus *Meseres* Schewiakoff, 1892 (1 species)
Subclass Oligotrichia Bütschli, 1887 (139 aloricate species)
Order Oligotrichida Bütschli, 1887** = oligotrichids** (103 species)
Family Tontoniidae Agatha, 2004** = tontoniids** (11 species)
Genus *Laboea* Lohmann, 1908 (1 species)
Genus *Paratontonia* Jankowski, 1978 (3 species)
Genus *Pseudotontonia* Agatha, 2004 (2 species)
Genus *Spirotontonia* Agatha, 2004 (3 species)
Genus *Tontonia* Fauré-Fremiet, 1914 (2 species)
Family Cyrtostrombidiidae Agatha, 2004 (3 species)
Genus *Cyrtostrombidium* Lynn & Gilron, 1993 (3 species)
Family Pelagostrombidiidae Agatha, 2004 (0 species)
Genus *Limnostrombidium* Krainer, 1995 (0 species)
Genus *Pelagostrombidium* Krainer, 1991 (0 species)
Family Strombidiidae Fauré-Fremiet, 1970 (89 species)
Genus *Apostrombidium* Xu, Warren & Song, 2009 (1 species)
Genus *Foissneridium* Agatha, 2010 (1 species)
Genus *Novistrombidium* Song & Bradbury, 1998 (3 species)
Genus *Omegastrombidium* Agatha, 2004 (3 species)
Genus *Opisthostrombidium* Agatha, 2010 (2 species)
Genus *Parallelostrombidium* Agatha, 2004 (3 species)
Genus *Spirostrombidium* Jankowski, 1978 (10 species)
Genus *Strombidium* Claparède & Lachmann, 1859 (65 species)
Genus *Varistrombidium* Xu, Warren & Song, 2009 (1 species)
Order Choreotrichida Small & Lynn, 1985** = choreotrichids** (36 aloricate species)
Family Leegaardiellidae Lynn & Montagnes, 1988 (3 species)
Genus *Leegaardiella* Lynn & Montagnes, 1988 (3 species)
Family Lohmanniellidae Montagnes & Lynn, 1991 (2 species)
Genus *Lohmanniella* Leegaard, 1915 (2 species)
Family Lynnellidae Liu, Yi, Lin & Al-Rasheid, 2011 (1 species)
Genus *Lynnella* Liu, Yi, Lin & Al-Rasheid, 2011 (1 species)
Family Strobilidiidae Kahl in Doflein & Reichenow, 1929 (12 species)
Genus *Pelagostrobilidium* Petz, Song & Wilbert, 1995 (4 species)
Genus *Rimostrombidium* Jankowski, 1978 (8 species)
Genus *Strobilidium* Schewiakoff, 1892 (0 species)
Family Strombidinopsidae Small & Lynn, 1985 (18 species)
Genus *Parastrombidinopsis* Kim et al., 2005 (2 species)
Genus *Parastrombidium* Fauré-Fremiet, 1924 (1 species)
Genus *Strombidinopsis* Kent, 1881 (15 species)

The first ciliate assigned to the oligotrichids was the marine species *Strombidium sulcatum* Claparède and Lachmann, 1859 [1]. The freshwater species *Strombidion caudatum* Fromentel, 1876 [2] is the first known aloricate choreotrichid ciliate, as it was transferred to the genus *Strobilidium* by Foissner [3]. In 1773, Müller described the first halteriid, viz., the freshwater species *Trichoda grandinella* [4], which was affiliated with the genus *Halteria* by Dujardin [5].

**Figure 1:**
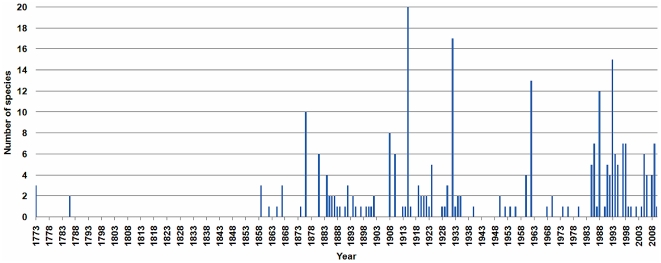
Rate of discovery. The published descriptions of new halteriid, oligotrichid, and aloricate choreotrichid species (including synonyms, nomina dubia, and nomina oblita) per year.

In their revisions, Kent listed 21 species [6], Awerinzew 10 species [7], and Kahl 84 species [8],[9], while the most recent monographs published in 1985 and 1986 [10], [11] considered 127 species of aloricate Oligotrichea. The rate of discovery (Figure 1) reflects the introduction and improvement of light microscopy (period ∼1860–1960) for the study of live and preserved specimens and the introduction of cytological staining methods (i.e., protargol and silver nitrate impregnation; period ∼1980–present) to reveal the ciliary pattern and nuclear apparatus. The rate of discovery was also distinctly influenced by the trend to neotypify species rather than to establish new ones, assuming that the majority of species has a cosmopolitan distribution [12]–[14]. Accordingly, the intensity of taxonomic studies was much higher during the past thirty years than implied by Figure 1. Currently, species descriptions do not only comprise information from live observation, silver impregnation, and scanning electron microscopy, but often also sequence data of the small subunit ribosomal RNA gene (SSrRNA).

### Ecology and Environments

Ciliates are highly developed unicellular eukaryotes, which propagate mainly asexually by transverse fission. The oligotrichids and choreotrichids are ciliate taxa episodically dominating the marine microzooplankton with maximum abundances of up to 1.2×10^6^ individuals per litre in the upper water layers [15]–[18]. Such high cell densities (“blooms”) might cause water colourations [15], [19]. The halteriids, however, occur only rarely and with low abundances in marine and brackish sea water. While the majority of Oligotrichea has a planktonic life style, 17 aloricate species are closely associated with the marine benthal, possessing special ciliary structures (thigmotactic membranelles) for a temporary attachment and/or migration on the substrate [8], [20], [21], and three species are endocommensals in sea urchins (see ‘Results’; [22]–[24]).

Most genera occur exclusively in marine and brackish sea water: the oligotrichid genera *Apostrombidium*, *Cyrtostrombidium*, *Foissneridium*, *Laboea*, *Novistrombidium*, *Omegastrombidium*, *Opisthostrombidium*, *Parallelostrombidium*, *Paratontonia*, *Pseudotontonia*, *Spirostrombidium*, *Spirotontonia*, *Tontonia*, and *Varistrombidium*, and the choreotrichid genera *Leegaardiella*, *Lohmanniella*, *Lynnella*, *Parastrombidinopsis*, *Parastrombidium*, and *Strombidinopsis*. The oligotrichid genus *Strombidium*, the choreotrichid genera *Rimostrombidium* and *Pelagostrobilidium*, and the halteriid genera *Meseres* and *Pelagohalteria* embrace both marine and freshwater species. The halteriid genus *Halteria*, the oligotrichid genera *Limnostrombidium* and *Pelagostrombidium*, and the choreotrichid genus *Strobilidium* are apparently restricted to freshwater, as the few records from brackish or marine environments are probably based on misidentifications (Table 1). All in all, there are 29 freshwater-specific species and six with a distribution only in saline inland waters or lakes (including the Caspian Sea), which are excluded from this compilation.

The Oligotrichea are members of the multi-step microbial food web. They mainly ingest bacteria as well as autotrophic and heterotrophic nanoplankton (2–20 µm) and are preyed upon by a wide variety of planktonic metazoans (e.g., copepods, fish larvae; [25]–[27]). Hence, they contribute to the energy flux of the conventional phytoplankton-based planktonic food web and may change the community composition of the bacterioplankton and nanoplankton through selective feeding [28]. Most/many oligotrichids are mixotrophic, “farming” plastids of their ingested algal prey and benefiting from the photosynthetic products [29]–[36].

Resting cysts, dormant stages formed during periods of adverse environmental conditions, are known in only a few marine species due to their rare occurrence, sedimentation, and difficult identification [37]–[47]. Apparently, open water species follow a seasonal encystment-excystment cycle [39], [41], [48], whereas the oligotrichid tide-pool ciliate *Strombidium oculatum* demonstrates a circatidal behaviour, encysting before high tide and excysting during low tide [42]–[44], [46], [49]–[51]. The resting cysts are flask-shaped or spindle-shaped with a solid, hollow, or frothy plug closing the emergence pore. The surface of the cyst is smooth or may bear spines variable in number and length.

### Species Diversity

Ciliates have complex morphologies, which can be revealed by cytological staining techniques and electron microscopy (see ‘Morphology’). While fossils of tintinnids (loricate Oligotrichea) reach back to the Ordovician or possibly even Mesoproterozoic era [52], there are no remains of the probably older aloricate Oligotrichea. The number of reliable species in marine and brackish sea water amounts to 103 in the 15 oligotrichid genera, 36 in the eight aloricate choreotrichid genera, and two in the two halteriid genera (Table 1 and 1), based on my revision of the aloricate Oligotrichea in preparation.

### Classification and Phylogeny

The Oligotrichea are separated from the closely related hypotrich and stichotrich spirotrichs (e.g., *Euplotes*, *Stylonychia*, *Oxytricha*) by several apomorphies: (i) a globular to obconical cell shape; (ii) a planktonic life style; (iii) an apically located adoral zone of membranelles (fan-like units composed of densely spaced cilia); (iv) a bipartition of the adoral zone in a collar portion with large membranelles and a buccal portion with small membranelles; (v) a lack of cirri (bristle-like complexes of somatic cilia); and (vi) an enantiotropic division mode (an inverse orientation of mother/proter and daughter/opisthe). According to cladistic analyses and phylogenies of the SSrRNA gene, the oligotrichids and choreotrichids are monophyletic [37], [53]–[64]. Concerning the position of the halteriids, however, the morphologic and genetic data are inconsistent, i.e., the morphology and pattern of cell division indicate a sister group relationship with a cluster formed by the oligotrichids and choreotrichids, whereas the halteriids are located among the stichotrichs (frequently as an adelphotaxon of *Oxytricha granulifera*) and thus distinctly apart from the oligotrichids and choreotrichids in the molecular trees [61], [65]–[67].

The hypothesized evolution of the somatic ciliary patterns is one of the main feature complexes integrated into the cladistic analyses [53], [54], [68], [69]. Agatha [53], [68] assumed a convergent development of the ciliary patterns in the tailless oligotrichids and the tailed tontoniids, as the contractile tail is considered a strong synapomorphy due to its complex and unique ultrastructure. However, gene sequence analyses indicated that the sinistrally spiralled ciliary pattern is a synapomorphy of the tailed genus *Spirotontonia* and the secondarily tailless monotypic genus *Laboea*. Otherwise, the morphologic tree of the oligotrichids matches rather well the SSrRNA phylogenies [69]. In both kind of trees, the aloricate choreotrichids are paraphyletic, but differ in the position of the genus *Parastrombidinopsis*. According to its morphology, it is an adelphotaxon of the genus *Strombidinopsis* at the base of the choreotrichids, whereas it represents a sister group to the more highly developed tintinnids in the molecular trees [54], [58].

### Morphology

In contrast to the majority of eukaryotes, ciliates are heterokaryotic, possessing two kinds of nuclei: one, rarely two or more diploid micronuclei involved into the sexual processes (conjugation) and usually one or several highly polyploid macronucleus nodules mainly controlling the metabolism. The aloricate Oligotrichea are globular, subspherical, ellipsoidal, obconical, or obovoidal and measure 15–260 µm. The somatic ciliature is often reduced, whereas the conspicuous adoral zone of membranelles at the apical cell end is used for locomotion and food collection by filter feeding [70].

**Figure 2:**
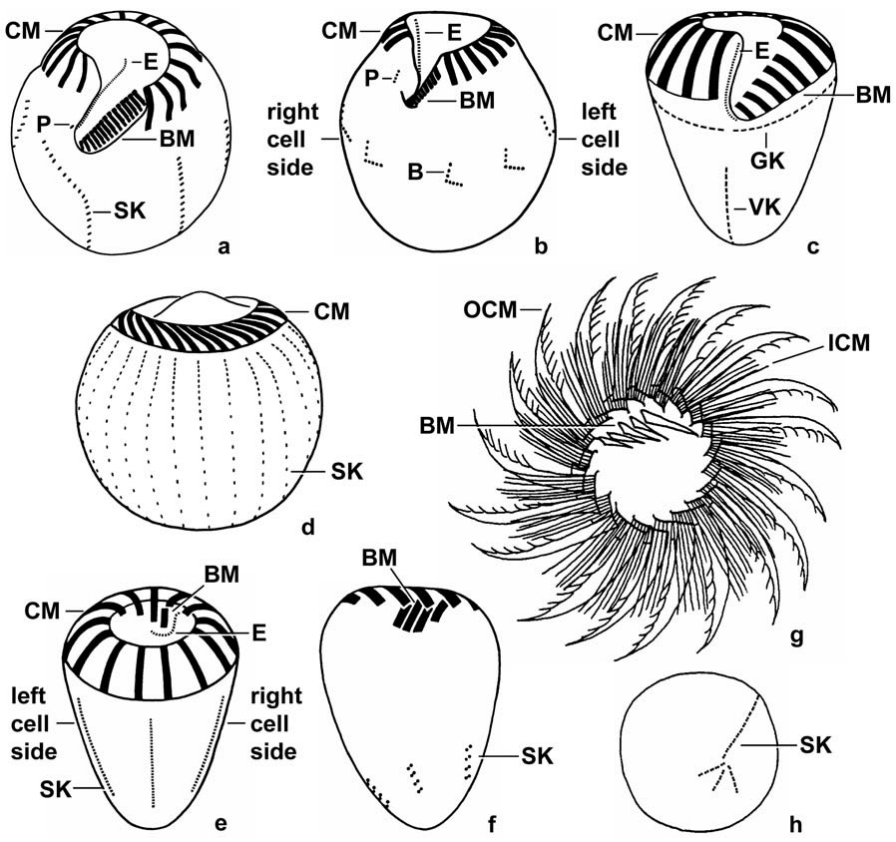
Morphology of main groups. Generalized ventral (a–c, f), dorsal (d, e), top (g), and posterior polar (h) views after protargol impregnation (a, after [190]; b, d, f, originals; c, e, after [53]; g, h, after [192]). **a, b:** The halteriid genera *Meseres* and *Pelagohalteria*. **c:** The oligotrichid genus *Strombidium*. **d–h:** The choreotrichid genera *Strombidinopsis* (d), *Rimostrombidium* (e), *Lohmanniella* (f), and *Leegaardiella* (g, h). B – bristle kineties, BM – buccal membranelles, CM – collar membranelles, E – endoral membrane, GK – girdle kinety, ICM – inner portion of collar membranelles, OCM – outer portion of collar membranelles, P – paroral membrane, SK – somatic kineties, VK – ventral kinety.

Halteriids (Figures 2a, b). In the halteriids, the adoral zone of membranelles is C-shaped with a distinct ventral gap and consists of a collar portion with large membranelles and a buccal portion with small membranelles. On the inner wall of the buccal lip, a longitudinal row of occasionally ciliated basal bodies, the endoral membrane, extends into the eccentric buccal cavity. The few ciliated basal bodies on the outer cell surface at the level of the cytostome represent the paroral membrane. In the genus *Meseres*, the somatic ciliature is arranged in several longitudinal rows composed of dikinetids (paired basal bodies), each with a cilium associated only with the anterior basal body (Figure 2a). In *Pelagohalteria*, the short and equatorially arranged somatic kineties consist of a longitudinal anterior and a horizontal posterior portion (Figure 2b). Its somatic cilia are conspicuously long and form bristles. Usually, the specimens rotate on the spot, interrupted by long jumps.

Oligotrichids (Figure 2c). In the oligotrichids, the adoral zone of membranelles is C-shaped with a distinct ventral gap and consists of a collar portion with large membranelles and a buccal portion with small membranelles. On the inner wall of the buccal lip, a longitudinal row of occasionally ciliated basal bodies, the endoral membrane, extends into the eccentric buccal cavity. The somatic ciliature typically comprises two ciliary rows: the girdle kinety and the ventral kinety. These kineties are composed of dikinetids, each with a stubby cilium associated only with the left or anterior basal body. Whereas the girdle kinety is arranged in several patterns, the ventral kinety generally extends longitudinally in the posterior cell portion. Rod-shaped or needle-shaped extrusomes (extrusive organelles) are usually attached to the cell membrane directly anteriorly to the girdle kinety and extend obliquely into the cytoplasm [71]. The cell cortex posterior to the girdle kinety typically contains a layer of polygonal polysaccharide platelets [31], [60], [72]–[74]. The specimens usually swim in spirals by rotation about their main cell axis.

Choreotrichids (Figures 2d–h). In the choreotrichids, the adoral zone of membranelles forms a circle. The large collar membranelles insert on an elevated rim around the peristomial field. Some of them are elongated, extending into the eccentric buccal cavity, which also contains the small buccal membranelles. In the genera *Lynnella*, *Parastrombidinopsis*, and *Parastrombidium*, however, the adoral zone of membranelles opened secondarily, producing an indistinct ventral gap. The genus *Leegaardiella* is exceptional in its bipartited bases (polykinetids) of the collar membranelles, i.e., they consist of an outer portion with long cilia and an inner portion with short cilia (Figure 2g). While the structure of the somatic ciliature is rather uniform in the oligotrichids, the choreotrichids show a wide variety of patterns: (i) in the genera *Pelagostrobilidium* and *Rimostrombidium*, the stubby cilia are very densely arranged in a few longitudinal or curved monokinetidal (composed of single basal bodies) rows and bent leftwards by cytoplasmic flaps (kinetal lips) covering their bases (Figure 2e); (ii) in the genera *Strombidinopsis*, *Parastrombidinopsis*, and *Parastrombidium*, the somatic ciliature is well developed and comprises several longitudinal rows composed of dikinetids, each with two cilia (Figure 2d); (iii) in the genus *Lohmanniella*, the few kineties are short, extending in the posterior cell portion, and consist of dikinetids, each with a cilium associated only with the posterior basal body (Figure 2f); (iv) in the genus *Leegaardiella*, the few kineties are short, extending in the posterior cell portion, and consist of dikinetids, each with two cilia or one cilium associated only with the anterior basal body (Figure 2h); and (v) in the genus *Lynnella*, one kinety is monokinetidal, while the other consists of dikinetids, each with a cilium associated only with the posterior basal body. Many aloricate choreotrichids are able to jump.

## Materials and Methods

### Data Source

While the biogeography of tintinnids (loricate Oligotrichea) is comparatively well studied [75]–[79], a census and survey on the global distribution of aloricate Oligotrichea are not available. Even in recent estimations of marine species richness, ciliates are not considered or are subsumed under the protists [80], [81]. A preliminary list of marine aloricate Oligotrichea merely exists for European sea regions [82].

The present compilation is based on hundreds of taxonomical and ecological studies, considering the accepted species mentioned (Table S1) and their synonyms; however, it cannot be excluded that some ecological papers might have been overlooked. The available records were classified according to their quality: (i) reliable records from the type or neotype locality accompanied by the original description or redescription; (ii) more or less reliable records supported by descriptions, measurements, and/or illustrations; and (iii) unsubstantiated records based on uncertain identifications.

### Biogeographic Subdivisions

The present analysis of the global distribution is not restricted to the pelagial, benthal, and sea ice of marine waters, but also includes all records from brackish sea waters in estuaries, fjords, coastal lagoons, the Baltic Sea, and the Black Sea. Finally, seven regions of the oceans were distinguished (Table S1), whose limits roughly correspond with the latitudinal-physical geographic zonation of water masses proposed by Van der Spoel and Heyman [83]: the Arctic and Subarctic waters are pooled; the northern temperate, northern subtropical, and northern tropical waters are united each in the North Atlantic and North Pacific; the southern tropical, southern subtropical, and southern temperate waters are lumped each in the South Atlantic, South Pacific, and Indian Ocean; and the Subantarctic and Antarctic waters are amalgamated. Furthermore, the Mediterranean, Baltic, and Black Sea are considered. Even though almost all studies were performed in neritic waters, the recorded species are supposed to occur also in the affiliated oceanic regions.

## Results

In spite of the comprehensive literature research, the species inventories presented in Table S1are fragmentary and influenced by various limitations (see below) preventing detailed comparisons and ecological analyses.

The majority of aloricate Oligotrichea (94 morphospecies) has a wide, occasionally cosmopolitan distribution (occurring in all oceans and seas from the Arctic through the tropics to the Antarctic), while 47 morphospecies are restricted to single geographic regions and include, conservatively estimated, 12 endemic morphospecies. The choreotrichid *Leegaardiella elbraechteri* and the oligotrichids *Spirostrombidium echini* (possibly specific to its sea urchin host), *Strombidium glaciale*, *S. kryale*, *S. syowaense* nom. corr. (specific epithet emended, as the genus name is neuter gender), and *Tontonia antarctica* were only found in the Antarctic Sea. The choreotrichid *Rimostrombidium sulcatum* as well as the oligotrichids *Strombidium costatum* and *S. opisthostomum* are possibly confined to the Black Sea. The oligotrichids *Strombidium foissneri* and *S. rapulum* are restricted to the North Pacific, possibly due to the geographic ranges of their sea urchin hosts. Among the endemics, the choreotrichid *Strombidinopsis cercionis* represents a “flagship” species in the sense of Tyler [84]. Because of its unique shape (pyriform with a posterior spine ∼30 µm long) the species is so conspicuous that it would have probably been recorded if it indeed occurred outside the Caribbean Sea. However, it cannot be excluded that *S. cercionis* is a young species that not yet fully explored its potential range. So, endemic species occur within widely distributed genera of aloricate Oligotrichea, as in tintinnids [75]. The oligotrichid *Parallelostrombidium rhyticollare* displays like the tintinnid *Acanthostomella norvegica*, the dinoflagellate *Polarella glacialis*, and some planktonic foraminifera a bipolar distribution, which possibly results from the glaciation during the Neogene [75], [85]–[87]. The other species recorded only in a certain region might actually have a wider distribution (see below).

**Figure 3:**
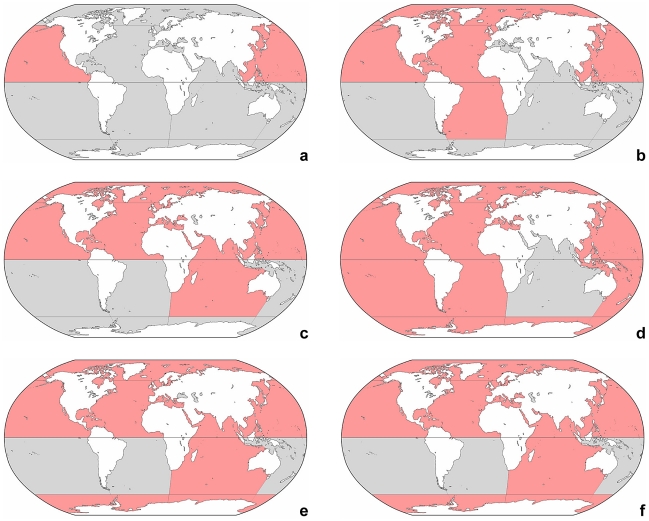
Global distribution of genera. Red colour marks the presumptive range in marine and brackish sea water (Table S1). Note that the distribution in freshwater and saline inland waters is not considered. **a:** *Apostrombidium*, *Lynnella*, *Opisthostrombidium*, and *Parastrombidinopsis*. **b:** *Cyrtostrombidium*. **c:** *Foissneridium*. **d:** *Laboea*. **e:** *Leegaardiella*. **f:** *Lohmanniella* and *Rimostrombidium*.

**Figure 4:**
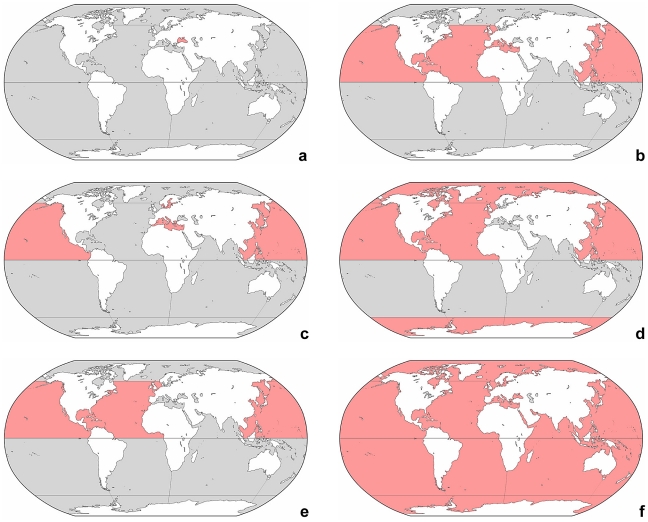
Global distribution of genera. Red colour marks the presumptive range in marine and brackish sea water (Table S1). Note that the distribution in freshwater and saline inland waters is not considered. **a:** *Meseres*. **b:** *Novistrombidium*. **c:** *Omegastrombidium*. **d:** *Parallelostrombidium*. **e:** *Parastrombidium*. **f:** *Paratontonia* and *Strombidium*.

**Figure 5:**
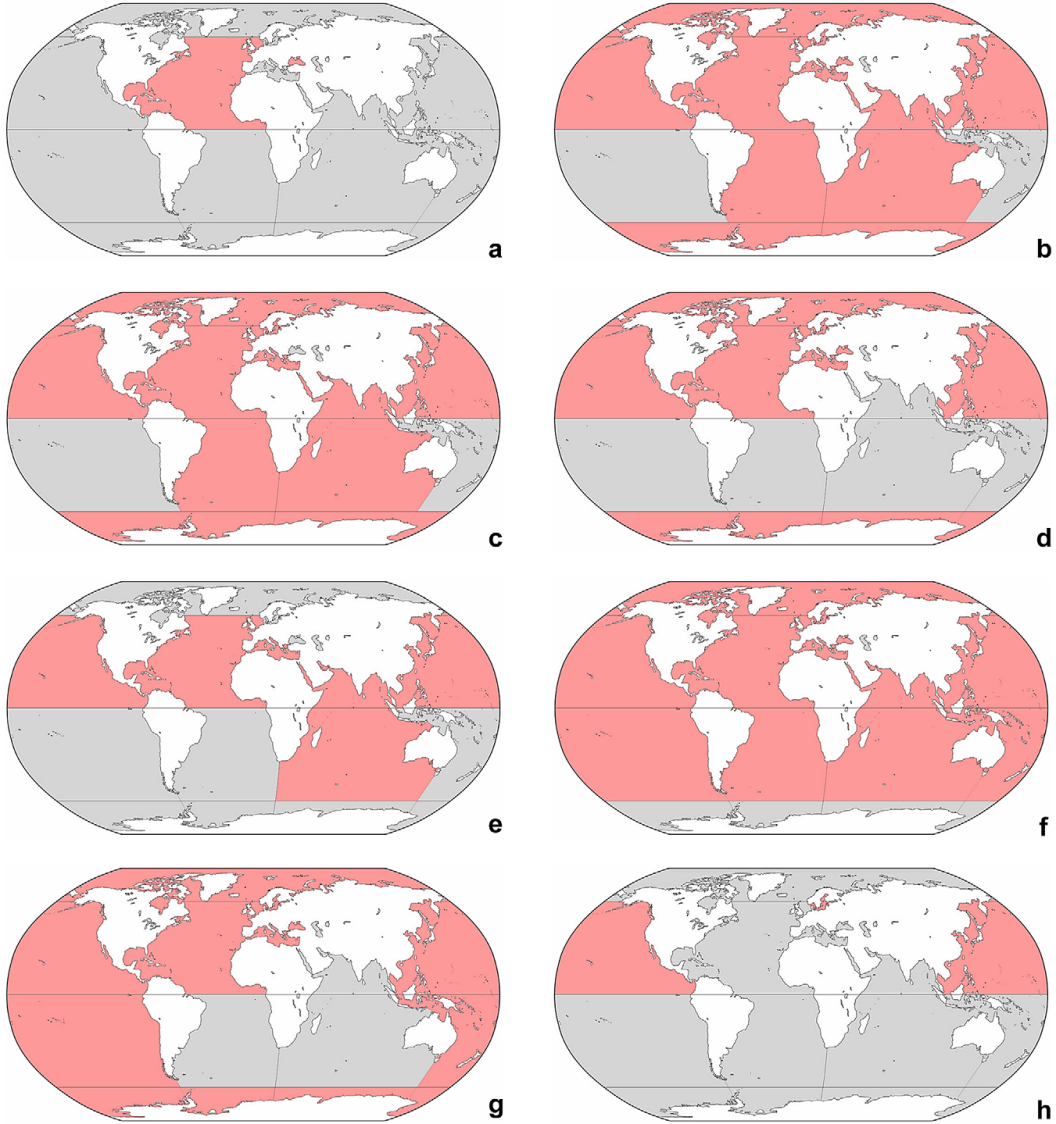
Global distribution of genera. Red colour marks the presumptive range in marine and brackish sea water (Table S1). Note that the distribution in freshwater and saline inland waters is not considered. **a:** *Pelagohalteria*. **b:** *Pelagostrobilidium*. **c:** *Pseudotontonia*. **d:** *Spirostrombidium*. **e:** *Spirotontonia*. **f:** *Strombidinopsis*. **g:** *Tontonia*. **h:** *Varistrombidium*.

The distribution of the genera presented here is like that of the species preliminary and necessitates more detailed studies. Several oversimplified geographic patterns are recognizable: (i) a cosmopolitan distribution in *Laboea*, *Leegaardiella*, *Lohmanniella*, *Parallelostrombidium*, *Paratontonia*, *Pelagostrobilidium*, *Pseudotontonia*, *Rimostrombidium*, *Spirostrombidium*, *Strombidium*, and *Tontonia* (Figure 3d–f, 4d, f, 5b–d, g); (ii) a worldwide distribution with the exception of the Antarctic Sea in *Strombidinopsis* (Figure 5f); (iii) a distribution roughly restricted to the northern hemisphere in *Cyrtostrombidium* and *Foissneridium* (Figure 3b, c); (iv) a distribution only in the northern hemisphere with the exception of the Arctic Sea in *Novistrombidium*, *Omegastrombidium*, *Parastrombidium*, *Spirotontonia*, and *Varistrombidium* (Figure 4b, c, e, 5e, h); (v) a distribution confined to the North Pacific in *Apostrombidium*, *Lynnella*, *Opisthostrombidium*, and *Parastrombidinopsis* (Figure 3a); (vi) a distribution limited to the North Atlantic, Black Sea, and probably Mediterranean in *Pelagohalteria* (Figure 5a); and (vii) a distribution restricted to the Black Sea in *Meseres* (Figure 4a), which mainly occurs in freshwater. The choreotrichid *Lohmanniella oviformis* and the oligotrichids *Laboea strobila*, *Paratontonia gracillima*, *Strombidium conicum*, *S. dalum*, and *S. sulcatum* have a worldwide range, although subtle morphologic differences indicate the presence of a species complex at least in *Strombidium sulcatum*.

The number of accepted morphospecies ranges from nine for the South Pacific to 95 for the North Atlantic (Table S1). A high diversity of aloricate Oligotrichea is also found in the North Pacific (94 species), Mediterranean (48 species), and Black Sea (46 species). Generally, the recorded species richness is lower in the southern hemisphere than in the northern (see below).

## Discussion

### Limitations

The data on the biogeography and diversity of aloricate Oligotrichea are strongly influenced by taxonomy and investigation methods. Furthermore, the natural patterns have been disturbed by human impact.

- Taxonomy. The accurate circumscription of species is an essential requirement for biodiversity and biogeography assessments. Traditionally, morphological traits were used to define and identify ciliate species, viz., the morphospecies concept was employed. Taxonomic mistakes and uncertainties, e.g., the tendency to identify specimens from new regions with European species despite subtle differences [88] and unjustified synonymizations, affect the diversity and geographic ranges perceived: taxonomic separations that mainly concern sympatric populations cause a higher global and local species diversity, while separations of allopatric populations on species level result in a decreasing relative local species richness due to a higher proportion of endemics [89]. Recent molecular and ecological studies showed that the morphospecies concept is too conservative in several groups of marine plankton protists, i.e., a tremendous genetic diversity indicating cryptic (no morphologic differences in cell and resting cyst) or pseudocryptic (subtle morphologic differences) species is frequently hidden within a morphospecies [90]–[96]. The discovery of these distinct (cut-off divergence of 1–2%) haplotypes underlying the morphospecies contributes to an increase of the perceived total taxonomic diversity. Simultaneously, the geographic ranges are probably reduced and endemic haplotypes/biological species might become evident [97].
- Investigation methods. Most species-specific features of the ciliates are recognizable in vivo and after cytological stainings (silver impregnation techniques). However, the application of both methods requires training by an expert. Since the Lugol-fixed material frequently used in ecological studies does not provide these characteristics, the discovery of new species and the identification of known ones is hardly feasible in such material. Furthermore, the fixation techniques are selective [98]. Generally, a higher taxonomic resolution results in a higher species diversity and a lower relative local species richness [99]. However, the species richness recorded in a certain region also depends on the spatial and temporal resolution of the sampling [99]–[101]. Hence, thorough studies are able to unveiled large portions of the otherwise cryptic diversity (organisms that are not detected as they are rare and/or patchily distributed) in marine protists, enhancing the species richness and influencing the number of endemics. Since ciliate taxonomists show a patchy distribution with “hot spots” of taxonomic excellence, comprehensive and reliable species lists are usually only available for the respective sampling regions.
- Human impact. The distribution patterns observed nowadays are the result of natural processes and anthropogenic influences, e.g., the more than thousand years of shipping with the transport of organisms by ballast water and the construction of canals connecting oceans and hence enabling the exchange of organisms [102]–[104].

### Biodiversity and Biogeography

The nature and extent of microbial biodiversity, biogeography, and community structure are controversially discussed: cosmopolitan vs. moderate endemicity model and niche-based vs. neutral theories [100], [105]–[109].

Baas Becking postulated that in microorganisms, including ciliates, “everything is everywhere, but the environment selects” [110]. According to the tenet of Finlay and colleagues, microorganisms have a high dispersal potential due to their small body size and huge abundance [105]. Therefore, marine plankton ciliates should show a ubiquitous dispersal (random across all spatial scales), causing a cosmopolitan distribution wherever the required habitat is realized, a low allopatric speciation, a low global but high local diversity, and low extinction rates. The huge abundances are the fundamental drivers of the ubiquitous dispersal of encysted and active microorganisms [111]. However, there are only few very abundant species of aloricate Oligotrichea in the plankton, while most species are only moderately abundant or even very rare [106], [112]–[118]. Additionally, the transport by ocean currents is non-random, resulting in an uneven distribution [77]–[79], [93], [119]. A ubiquitous dispersal is further hampered by the duration of the transport by ocean currents, i.e., a global circulation needs more than 1000 years [86], [120] and even the transport by the Gulf Stream from the east coast of the USA to European coastal waters lasts about two years [121], [122]. Active forms do not tolerate the uncomfortable conditions encountered during such long-distance dispersal (e.g., low temperatures and starvation in winter), and resting cysts are rarely formed by marine plankton ciliates, as indicated by molecular analyses comparing plankton and benthos samples from the same coastal sites [123]. Since the cysts are also at risk to sediment (14.4 m d^−1^ in freshwater oligotrichids; [41], [124]) and the excystment ability decreases from ∼60% to an incapability after some month at low temperatures ( = conditions met in deep water layers; [40], [48], [125]), a long-distance dispersal of the cysts and a subsequent population growth are thus apparently rare events. The isolation caused by distance [86], [126], patchiness [127] due to the heterogeneity of the oceans [128], [129], front systems, changes in the ocean circulation patterns, vicariance events, glacial-interglacial climate dynamics, and global extinction events might have fostered together with the short generation times [106], [117] allopatric speciation in aloricate Oligotrichea. Furthermore, there is evidence for parapatric and sympatric speciation in some planktonic protists [91], [130]–[134]. Indeed, the statistical analyses of ciliate communities from the freshwater pelagial and marine benthal performed by Hillebrand and co-authors refuted the prediction that unicellular organisms generally have a higher relative local species diversity than metazoa [89]; thus, they must not necessarily be ubiquitous, but at least some species might possess a biogeography. Actually, geographic patterns are recognizable in many species or haplotypes of marine plankton protist (e.g., foraminifera, radiolaria, dinoflagellates, tintinnids, and aloricate Oligotrichea; [this study], [ 83], [91]–[93], [95], [135]–[140]) as well as in many macrozooplankton species [96]. The moderate endemicity model attenuates the “everything is everywhere” tenet, suggesting that (i) the abundances and thus the migration rates are low in ∼90% of the species, (ii) the extinction rates are moderate, (iii) the proportion of the global species pool found locally is moderate, and (iv) ∼30% of the species are endemics [117]. Consistent with this model and the findings in the closely related tintinnids [75], the majority of aloricate Oligotrichea has a wide, possibly cosmopolitan distribution, while ∼33% of the morphospecies are restricted to certain geographic regions, and at least ∼9% of the morphospecies are endemic according to conservative estimations.

Molecular biogeography is still in its infancy in aloricate Oligotrichea. The few studies available focussed on the small subunit ribosomal RNA (SSrRNA) gene and/or the internal transcribed spacer (ITS) sequence from comparatively limited geographical samples. A gene flow was found between (i) the northwest Atlantic [115] and northwest Pacific [141], (ii) the Mediterranean [60] and northwest Pacific [56], [57], [142], and (iii) the Mediterranean [37], [55] and northwest Atlantic [61], [115], [116]. On the other hand, there are deviations in morphospecies from the northwest Pacific [56], [57] and northwest Atlantic (five SSrRNA nucleotides; [45]) and from the Mediterranean [60] and northwest Pacific (1.2% in the SSrRNA gene and in morphologic details; [142]). In morphologically similar freshwater halteriids, the conspicuous genetic diversity registered by Katz and colleagues was correlated with differences in the resting cysts (cyst species; [143]) and minute deviations in the cell morphology [144] indicating biological species. Hence, the genetic diversity within a morphospecies might at least partially result from an insufficient taxonomic resolution, viz., haplotypes which are indistinguishable in live and Lugol-fixed material at low (400–600×) magnification might be differentiated by an experienced morphological taxonomist, using live observation, silver impregnation, and light microscopy at high (1000×) magnification. For example, the analyses of the ITS regions indicated a huge genetic diversity in similar-sized tide-pool oligotrichids identified with *Strombidium oculatum* due to their green sequestered plastids and the prominent eye-spot in the apical protrusion [144]. It finally turned out that one haplotype had a >99% identity in the SSrRNA gene with *Strombidium apolatum* and one represents *Strombidium rassoulzadegani* [45], while it is still unknown whether one of the six further haplotypes really corresponds with *Strombidium oculatum*. So, the genetic diversity observed was mainly due to a lumping of species, which are morphologically distinct in live and silver-impregnated material (with or without a conspicuous posterior spine; girdle kinety continuous or with a distinct ventral gap; position of the extrusome girdle). This example is a plea for submitting molecular data of named species only after detailed morphological investigations of live and silver-impregnated specimens [145] and the deposition of permanent voucher slides in a recognized museum. Recent studies on a freshwater halteriid and aloricate choreotrichid suggested that the ecophysiological diversity is not only considerably larger than the morphological, but also tops that of the haplotypes [146], [147].

### Community Structure

The high diversity of plankton organisms, especially, protists, raised the question of “how a number of species can coexist in a relatively isotropic or unstructured environment all competing for the same sorts of materials” (“paradox of the plankton”; [148]). Not only extrinsic (weather-driven) fluctuations, as suggested by Hutchinson [148], but also intrinsic mechanisms within the plankton communities can fuel non-equilibrium dynamics and result in a coexistence of many species on a handful resources [149]–[151]. A further explanation for the diversity of similar species is provided by Hubbell's “neutral community model” [108]. The theory assumes that the interactions among species are equivalent on an individual ‘per capita’ basis. Since niche differentiation will, however, impact any of the basic processes of the neutral community model, Gravel and colleagues suggested the continuum hypothesis, reconciling both the niche-based and neutrality concepts [152]. Additionally, Alonso and co-authors emphasized that different mechanisms, although strictly violating the equivalence assumption, can also generate patterns resembling neutrality [153]. In aloricate Oligotrichea, the functional equivalence is limited, as they often show species-specific temperature optima for growth and threshold concentrations [154], [155] as well as salinity preferences [156], food selectivity [157]–[159], and different nutrition modes (see ‘Ecology and Environments’). Hence, the aloricate Oligotrichea are probably a physiologically quite heterogeneous group within the microbial food web. In tintinnids, the community structure was studied, using morphological approaches [112]–[114], whereas the investigation of marine aloricate Oligotrichea was either based on molecular data [115], [116] or morphological studies [160]. The tintinnid abundances in the southeast tropical Pacific Ocean usually fitted a log-series distribution coherent with the neutral community theory [112]. For Mediterranean tintinnids, the data are incoherent: while Sitran and colleagues found a log-normal distribution, indicating a strong impact of the environment [114], Dolan and co-workers observed a log-series distribution. A partition of the tintinnid assemblages revealed, however, a log-normal distribution in the core (numerical abundant) morphospecies and a log-series distribution in the occasional (rare) ones [113]. Similar findings were obtained for the core and occasional haplotypes in aloricate Oligotrichea from the east coast of the USA [115], [116]. Claessens and co-authors analyzed the ciliate community in the plankton of the Red Sea [160], [161]. The species abundances (in total ∼41% aloricate Oligotrichea, ∼37% tintinnids, and ∼22% other ciliate groups) most closely fitted the log-normal distribution mainly during mixing conditions and after onset of a stratification, while a log-series distribution was registered usually during the stratification. The authors concluded that the neutral community model did not explain the species diversity observed. Between-clone variation in the dominant cyanobacteria *Synechococcus* might be at least partially responsible for niche separation based on fine-scale food selectivity.

In general, the numbers of morphospecies found in the different geographic regions reflect rather the intensity of taxonomic research than the real diversity of aloricate Oligotrichea, viz., the North Atlantic, North Pacific, and Mediterranean harbour the largest numbers of accepted species (Table S1), which is consistent with the results of Bouchet [80]. These findings are also influenced by various further factors (see ‘Limitations’). Even though molecular analyses are able to screen large water volumes, there is evidence of a cryptic diversity [162]. Using a cut-off divergence of 1%, in sum 66 Oligotrichea haplotypes (including ∼12 tintinnids) were found in 50–60 litres of sea water, each taken at three distantly located sites along the east coast of the USA in spring and autumn. An estimation of the total haplotype diversity yielded a maximum of 325 haplotypes for a spring sample. In samples of 200 ml Lugol-fixed material taken on the same occasions, up to 19 morphotypes could be discerned under the light microscope at a magnification of 400–600× [115]. Due to the distinctly different volumes analyzed and the low taxonomic resolution provided by the Lugol-fixed material (see ‘Limitations’), the molecular and morphological data of this study are hardly comparable. In the Long Island Sound samples of this study, in sum 27 haplotypes of Oligotrichea (including at least three tintinnids) were found [115]. In 12 further samples of each two litres of sea water taken at the same site in summer, 62 additional haplotypes (including eight tintinnids) were recorded, while only five haplotypes from the former study were rediscovered [116]. This clearly shows the impact of the sampling effort on the diversity record. So, in total 89 Oligotrichea haplotypes (including tintinnids) were found in the Long Island Sound, which is similar to the number of morphologically identified species in the whole North Atlantic (95 without tintinnids; Table S1; [115], [116]). In total, 26 morphotypes of aloricate Oligotrichea were recorded in 6.6 litres formalin-fixed material taken at eleven stations in the Mediterranean at six different depths (1–100 m; [163]), whereas 48 morphospecies have been compiled for the whole region (Table S1). In the Arctic Sea, 38 morphologically identified species were recorded, but merely four oligotrichid sequences were found in 500–1000 ml of sea water, each taken in two depth, and a sediment sample at a single site near the west coast of Svalbard in summer (Table S1; [164]). Claessens and co-authors discovered 45 morphotypes of aloricate Oligotrichea in the Red Sea [160]. Since half of the morphotypes could not be identified, merely 25 species from the Indian Ocean plus the Red Sea are assembled in Table S1.

At the present state of knowledge, a differentiation of longitudinal trends and undersampling effects (indicated by the low numbers of species descriptions and redescriptions; Table S1) is impossible in the aloricate Oligotrichea. In planktonic foraminifera and tintinnids, however, the number of taxa increases from high to low latitudes with two peaks near the Tropics of Cancer and Capricorn (20–30°N and S, respectively; [165]–[167]).

In contrast to the tintinnids, there are only few regional inventories about aloricate Oligotrichea. Hence, it is infeasible to apply the same statistical approaches as in soil ciliates [100]. Nevertheless, the steady rate of species descriptions indicates a much higher total diversity. Foissner and colleagues concluded from habitat studies that the number of known ciliate species must be doubled [143]. “Cyst species” increase the number further by 50%, and “genetic species” will again double the number. So, the authors argued that eventually 83–89% of the ciliate diversity are unknown. A similar percentage was estimated by Costello and co-workers with 70–80% in marine species [81] and by the Convention on Biological Diversity with about 95% in protists (∼575,000 unknown species, as estimated from Figure 1 in [168]). Taking the conservative estimate of unknown species by Foissner and co-workers [143] and the data of the present study (Table S1), the number of biological species in aloricate Oligotrichea would amount to 560–860 for the North Atlantic and to 830–1280 worldwide.

### Significance of Aloricate Oligotrichea

The aloricate Oligotrichea are occasionally important grazers on phytoplankton [169]–[171] and thus possibly influence (i) the ocean acidification via sequestration of anthropogenic CO_2_ by the phytoplankton and (ii) the climate by the release of DMS (dimethylsulfide) that can act as cloud condensation nuclei [172], [173]. On the other hand, aloricate Oligotrichea supply the primary production of algae with inorganic nutrients through recycling [174]. Some species feed on harmful and bloom-forming plankton algae [175]–[181] and hence might control these toxic organisms that contaminate seafood and/or kill marine organisms, e.g., fish. Neutral communities (see above) are characterized by high functional redundancy; thus, the extinction of a few species should have little effect on the functional integrity of the whole community or ecosystem [111]. Even if (i) alternative nutritional strategies, such as mixotrophy, might have a stabilizing effect on ecosystem functioning and (ii) the rare species might compensate species loss due to dramatic shifts in the environmental conditions, the resulting changes in the community structure of aloricate Oligotrichea might affect higher trophical levels substantially [182], [183]. Accordingly, the aloricate Oligotrichea should not any longer be ignored in conservation issues [184].

### Future Research

Although many governments, through the Convention on Biological Diversity, have acknowledged the shortage of trained taxonomists and the Global Taxonomic Initiative was established to overcome this problem (“Taxonomic impediment”; [185]), the number of ciliate taxonomists is still too low (i) to describe the conservatively estimated 690–1140 new species only in the aloricate Oligotrichea living in marine and brackish sea water, (ii) to investigate the southern hemisphere, the oceanic regions, and the deep sea, and (iii) to identify the huge amount of gene sequences yielded by environmental samples. Since the recognition of new morphospecies largely depends on the availability of reliable identification guides, the production of regularly updated comprehensive monographs should be a priority [81].

Molecular analyses, using SSrRNA and ITS sequences, have become an affordable and practical method that is increasingly applied in diversity studies. However, species identification merely by molecules cannot compensate the disappearing taxonomic expertise, as it is fraught with the same constraints and inconsistencies plaguing morphological judgments of species limits [186]. A gene sequence that does not exactly match a previously sequenced and morphologically identified species cannot be assigned and thus determined, as a predictive rule about the degree of genetic divergence required for the recognition of distinct species is impossible [90], [186], [187]. Further, gene sequences of misidentified species prevent correct interpretations of phylogenetic trees and geographic ranges. Accordingly, the identification of sequenced specimens should be based on detailed studies of live and silver-impregnated material by a morphological taxonomist, and permanent slides should be deposited [188], [189].

Future taxonomic studies will certainly (i) provide morphological data from additional populations that contribute to a better circumscription of the known species, (ii) identify further taxonomically significant features, and (iii) discover new species. Genetic analyses of environmental samples might assist in the detection of these new species, especially, the rare ones. The taxonomic investigations should use live observation, silver impregnation techniques, and electron microscopy as in [190] and should be complemented by molecular studies not only of the SSrRNA and ITS sequences, but also of the cytochrome oxidase I gene (COI), which might reveal biogeographic patterns even in organisms with identical SSrRNA and ITS genes [191]. By means of systematic sampling, the species diversity and abundances from various distances and environmental conditions should be recorded to better distinguish between contemporary environmental and historical contingencies causing spatial variability [107]. Likewise, the ecological and evolutionary processes of speciation and the mechanisms by which diversity is maintained in the pelagic realm require further investigations. In order to attain these objectives, synergistic approaches combining the expertises of morphological and molecular taxonomists, ecologists, and physiologists are indispensable.

## Supporting Information

Table S1 Global distribution of halteriids (H), oligotrichids (O), and choreotrichids (C) in marine and brackish sea waters.
(DOC)
