# Supplementary material for: Global Diversity of Aloricate Oligotrichea (Protista, Ciliophora, Spirotricha) in Marine and Brackish Sea Water
Source: PLoS One. 2011 Aug 10;6(8):e22466. doi: 10.1371/journal.pone.0022466 (PMC3154192; doi:10.1371/journal.pone.0022466)
Supplement: Table S1 — Global distribution of halteriids (H), oligotrichids (O), and choreotrichids (C) in marine and brackish sea waters. (DOC) [file pone.0022466.s001.doc]

**Table S1.** Global distribution of halteriids (H), oligotrichids (O), and choreotrichids (C) in marine and brackish sea waters; the regions comprise the pelagial, benthal, and sea ice; + – unsubstantiated record; ++ – record substantiated by measurements, illustrations, and/or gene sequences; +++ – record from type or neotype locality with references in brackets.

| Taxon | Species | Antarctic Sea ^1^ | Arctic Sea ^2^ | Mediterranean ^3^ | North Atlantic ^4^ | South Atlantic ^5^ | North Pacific ^6^ | South Pacific ^7^ | Indian Ocean ^8^ | Baltic Sea ^9^ | Black Sea ^10^ |
| --- | --- | --- | --- | --- | --- | --- | --- | --- | --- | --- | --- |
| O | ***Apostrombidium*** |  |  |  |  |  | +++ |  |  |  |  |
| O | *Apostrombidium pseudokielum* |  |  |  |  |  | +++ [193] |  |  |  |  |
| O | ***Cyrtostrombidium*** |  | ++ |  | +++ | ++ | +++ |  |  |  |  |
| O | *Cyrtostrombidium boreale* |  | ++ |  |  |  | +++ [40] |  |  |  |  |
| O | *Cyrtostrombidium longisomum* |  |  |  | +++ [194] | ++ |  |  |  |  |  |
| O | *Cyrtostrombidium wailesi* |  |  |  | +++ [194] |  |  |  |  |  |  |
| O | ***Foissneridium*** |  | +++ | ++ | +++ |  | ++ |  | + | ++ | + |
| O | *Foissneridium constrictum* |  | +++ [195] | ++ | +++ [196] |  | ++ |  | + | ++ | + |
| O | ***Laboea*** | ++ | ++ | ++ | +++ | ++ | ++ | ++ |  | +++ | ++ |
| O | *Laboea strobila* | ++ | ++ | ++ | +++ [197] | ++ | ++ | ++ |  | +++ [198] | ++ |
| C | ***Leegaardiella*** | +++ | ++ | ++ | +++ |  | + |  | + | ++ |  |
| C | *Leegaardiella elbraechteri* | +++ [199] |  |  |  |  |  |  |  |  |  |
| C | *Leegaardiella ovalis* | + | ++ | ++ | +++ [192] |  | + |  | + |  |  |
| C | *Leegaardiella sol* | + | ++ | ++ | +++ [192] |  | + |  | + | ++ |  |
| C | ***Lohmanniella*** | +++ | ++ | ++ | +++ |  | ++ |  | ++ | ++ | + |
| C | *Lohmanniella glacicola* | +++ [199] |  |  |  |  | ++ |  |  |  |  |
| C | *Lohmanniella oviformis* | + | ++ | ++ | +++ [192, 200] |  | + |  | ++ | ++ | + |
| C | ***Lynnella*** |  |  |  |  |  | +++ |  |  |  |  |
| C | *Lynnella semiglobulosa* |  |  |  |  |  | +++ [201] |  |  |  |  |
| H | ***Meseres*** |  |  |  |  |  |  |  |  |  | + |
| H | *Meseres cordiformis* |  |  |  |  |  |  |  |  |  | + |
| O | ***Novistrombidium*** |  |  | +++ | ++ |  | +++ |  |  |  |  |
| O | *Novistrombidium orientale* |  |  |  |  |  | +++ [202] |  |  |  |  |
| O | *Novistrombidium sinicum* |  |  |  |  |  | +++ [202] |  |  |  |  |
| O | *Novistrombidium testaceum* |  |  | +++ [60, 203] | ++ |  | ++ |  |  |  |  |
| O | ***Omegastrombidium*** |  |  | ++ |  |  | +++ |  |  | ++ |  |
| O | *Omegastrombidium elegans* |  |  | ++ |  |  | +++ [204] |  |  | ++ |  |
| O | *Omegastrombidium jankowskii* |  |  |  |  |  | +++ [193] |  |  |  |  |
| O | *Omegastrombidium kahli* |  |  |  |  |  | +++ [193] |  |  |  |  |
| O | ***Opisthostrombidium*** |  |  |  |  |  | +++ |  |  |  |  |
| O | *Opisthostrombidium montagnesi* |  |  |  |  |  | +++ [205] |  |  |  |  |
| O | *Opisthostrombidium wilberti* |  |  |  |  |  | +++ [206] |  |  |  |  |
| O | ***Parallelostrombidium*** | +++ | ++ |  | + |  | +++ |  |  |  |  |
| O | *Parallelostrombidium paralatum* |  |  |  |  |  | +++ [205] |  |  |  |  |
| O | *Parallelostrombidium rhyticollare* | +++ [199, 207] | ++ |  |  |  |  |  |  |  |  |
| O | *Parallelostrombidium siculum* |  |  |  | + |  | +++ [208] |  |  |  |  |
| C | ***Parastrombidinopsis*** |  |  |  |  |  | +++ |  |  |  |  |
| C | *Parastrombidinopsis minima* |  |  |  |  |  | +++ [64] |  |  |  |  |
| C | *Parastrombidinopsis shimi* |  |  |  |  |  | +++ [58] |  |  |  |  |
| C | ***Parastrombidium*** |  |  |  | +++ |  | +++ |  |  |  |  |
| C | *Parastrombidium faurei* |  |  |  | +++ [209] |  | +++ [210] |  |  |  |  |
| O | ***Paratontonia*** | + | ++ | ++ | +++ | + | ++ | + | + | + | + |
| O | *Paratontonia gracillima* | + | + | ++ | +++ [209] | + | ++ | + | + | + | + |
| O | *Paratontonia mononucleata* |  |  |  | +++ [211] |  |  |  |  |  |  |
| O | *Paratontonia poopsia* |  | ++ |  | +++ [212] |  |  |  |  |  |  |
| H | ***Pelagohalteria*** |  |  |  | +++ |  |  |  |  |  | + |
| H | *Pelagohalteria cirrifera* |  |  |  | +++ [156] |  |  |  |  |  | + |
| C | ***Pelagostrobilidium*** | ++ | ++ | ++ | +++ | ++ | +++ |  | + | ++ | + |
| C | *Pelagostrobilidium epacrum* | + | ++ |  | +++ [192] | ++ |  |  |  |  | + |
| C | *Pelagostrobilidium neptuni* | ++ |  | ++ | ++ |  | +++ [208] |  | + |  |  |
| C | *Pelagostrobilidium simile* |  |  |  |  |  | +++ [213] |  |  |  |  |
| C | *Pelagostrobilidium spirale* |  | ++ | ++ | +++ [192, 200] |  | + |  | + | ++ | + |
| O | ***Pseudotontonia*** | + | + | ++ | +++ | ++ | +++ |  | + | ++ |  |
| O | *Pseudotontonia cornuta* | + |  | + | +++ [200] |  | +++ [214] |  | + | ++ |  |
| O | *Pseudotontonia simplicidens* |  | + | ++ | +++ [194] | ++ | ++ |  |  |  |  |
| C | ***Rimostrombidium*** | + | ++ | + | +++ |  | +++ |  | + | +++ | +++ |
| C | *Rimostrombidium caudatum* |  | ++ |  | +++ [215] |  | ++ |  |  | +++ [8] | + |
| C | *Rimostrombidium conicum* |  | + | + | +++ [8, 215] |  | ++ |  |  | ++ | + |
| C | *Rimostrombidium multinucleatum* | + | ++ |  | +++ [192] |  | ++ |  | + |  |  |
| C | *Rimostrombidium orientale* |  |  |  |  |  | +++ [213] |  |  |  |  |
| C | *Rimostrombidium sphaericum* |  |  |  | +++ [192] |  |  |  |  |  | + |
| C | *Rimostrombidium sulcatum* |  |  |  |  |  |  |  |  |  | +++ [216] |
| C | *Rimostrombidium undinum* |  |  |  | ++ |  | +++ [217] |  |  | ++ |  |
| C | *Rimostrombidium veniliae* |  |  |  | ++ |  | +++ [208] |  |  |  |  |
| O | ***Spirostrombidium*** | +++ | ++ | +++ | +++ |  | +++ |  |  | +++ | ++ |
| O | *Spirostrombidium agathae* |  |  |  |  |  | +++ [218] |  |  |  |  |
| O | *Spirostrombidium cinctum* |  |  |  | +++ [8] |  | +++ [219] |  |  | ++ | ++ |
| O | *Spirostrombidium echini* | +++ [220] |  |  |  |  |  |  |  |  |  |
| O | *Spirostrombidium oblongum* |  |  | +++ [221] |  |  |  |  |  | + |  |
| O | *Spirostrombidium platum* |  |  |  |  |  | +++ [222] |  |  |  |  |
| O | *Spirostrombidium pseudocinctum* | +++ [199] |  |  |  |  | +++ [223] |  |  |  |  |
| O | *Spirostrombidium pulchrum* |  | ++ | + | +++ [200] |  | + |  |  |  |  |
| O | *Spirostrombidium sauerbreyae* |  | + | + | ++ |  | + |  |  | +++ [224] | ++ |
| O | *Spirostrombidium schizostomum* |  |  |  | +++ [8] |  | +++ [218] |  |  |  |  |
| O | *Spirostrombidium urceolare* |  |  |  | ++ |  | +++ [225] |  |  | +++ [226] |  |
| O | ***Spirotontonia*** |  |  | ++ | ++ |  | +++ |  | + |  |  |
| O | *Spirotontonia grandis* |  |  | ++ | ++ |  | +++ [227] |  | + |  |  |
| O | *Spirotontonia taiwanica* |  |  |  |  |  | +++ [228] |  |  |  |  |
| O | *Spirotontonia turbinata* |  |  |  | ++ |  | +++ [213] |  |  |  |  |
| C | ***Strombidinopsis*** |  | +++ | +++ | +++ | ++ | +++ | + | + | ++ | ++ |
| C | *Strombidinopsis acuminata* |  | + | ++ | +++ [209, 229] |  | ++ |  | + | + | + |
| C | *Strombidinopsis batos* |  |  | + | ++ |  | +++ [229] |  |  |  |  |
| C | *Strombidinopsis cercionis* |  |  |  | +++ [229] |  |  |  |  |  |  |
| C | *Strombidinopsis chilorhax* |  |  |  | +++ [229] |  |  |  | + |  |  |
| C | *Strombidinopsis elegans* |  |  |  |  |  | +++ [213] |  |  |  | + |
| C | *Strombidinopsis elongata* |  |  |  | ++ | ++ | +++ [213] |  |  |  | + |
| C | *Strombidinopsis grandis* |  |  |  |  |  | +++ [230] |  |  |  |  |
| C | *Strombidinopsis jeokjo* |  |  |  | ++ |  | +++ [141] |  |  |  |  |
| C | *Strombidinopsis magna* |  |  |  | +++ [211] |  |  |  |  |  |  |
| C | *Strombidinopsis marina* |  | ++ |  | +++ [231] |  | + |  |  |  | + |
| C | *Strombidinopsis minima* |  | + | +++ [232, 233] | ++ |  | ++ |  | + | ++ | ++ |
| C | *Strombidinopsis multiauris* |  |  |  | + |  | +++ [208] |  |  |  | + |
| C | *Strombidinopsis pelagica* |  |  | + | +++ [209] |  |  | + |  |  | + |
| C | *Strombidinopsis sphaira* |  |  |  | +++ [229] |  | + |  | + |  |  |
| C | *Strombidinopsis spinifera* |  | +++ [229] |  | +++ [200] |  |  |  |  |  |  |
| O | ***Strombidium*** | +++ | +++ | +++ | +++ | ++ | +++ | + | ++ | +++ | +++ |
| O | *Strombidium acutum* | + | + | ++ | +++ [197, 200] | ++ | ++ |  |  |  | + |
| O | *Strombidium antarcticum* | +++ [199, 234] |  |  |  | + |  | + |  |  |  |
| O | *Strombidium apolatum* | +++ [235] |  |  | ++ |  | ++ |  |  |  |  |
| O | *Strombidium arenicola* |  |  | +++ [236] | +++ [237] |  | + |  |  |  | ++ |
| O | *Strombidium basimorphum* |  |  |  | ++ |  | +++ [217] |  |  |  |  |
| O | *Strombidium biarmatum* |  |  | +++ [37] | ++ |  |  |  |  |  |  |
| O | *Strombidium bilobum* |  |  |  | +++ [194] |  | + |  | + |  |  |
| O | *Strombidium calkinsi* |  | + |  | +++ [238] |  |  |  | ++ | + | ++ |
| O | *Strombidium capitatum* |  | ++ | ++ | +++ [197, 200] | ++ | ++ |  |  |  |  |
| O | *Strombidium chlorophilum* |  |  | + | +++ [197] |  |  |  |  |  |  |
| O | *Strombidium clavellinae* |  |  |  | +++ [239] |  |  |  |  |  |  |
| O | *Strombidium compressum* |  | + | ++ | +++ [196] |  | ++ |  |  | +++ [200] |  |
| O | *Strombidium conicum* | + | ++ | ++ | +++ [197] | ++ | ++ | + |  | +++ [198] | + |
| O | *Strombidium cornucopiae* |  |  | + | + |  | +++ [240] |  |  |  |  |
| O | *Strombidium coronatum* |  | ++ | ++ | +++ [200] |  | ++ |  | + | + | + |
| O | *Strombidium corsicum* |  |  | +++ [241] |  |  |  |  |  |  |  |
| O | *Strombidium costatum* |  |  |  |  |  |  |  |  |  | +++ [216] |
| O | *Strombidium crassulum* | +++ [199] | + | ++ | ++ | + | + | + |  | +++ [200] |  |
| O | *Strombidium dalum* | + | ++ | + | +++ [196] | ++ | + |  | + | ++ | + |
| O | *Strombidium emergens* | +++ [199] | + | ++ | ++ |  | ++ |  | + | +++ [200] | + |
| O | *Strombidium epidemum* |  |  | ++ | +++ [196] |  | + |  | + |  | + |
| O | *Strombidium eurystomum* |  |  |  | +++ [194] |  |  |  |  |  |  |
| O | *Strombidium faurei* |  | + |  | +++ [236] | ++ |  |  |  |  | + |
| O | *Strombidium filificum* |  |  |  | +++ [8] |  | + | + |  |  | + |
| O | *Strombidium foissneri* |  |  |  |  |  | +++ [23] |  |  |  |  |
| O | *Strombidium fourneleti* |  |  |  | +++ [236] |  |  |  |  |  |  |
| O | *Strombidium glaciale* | +++ [199] |  |  |  |  |  |  |  |  |  |
| O | *Strombidium globosaneum* |  |  |  |  |  | +++ [222] |  |  |  |  |
| O | *Strombidium grande* |  |  |  | + |  |  |  |  | +++ [242] |  |
| O | *Strombidium inclinatum* | + |  | ++ | ++ |  |  |  |  | +++ [243] | + |
| O | *Strombidium ioanum* |  |  |  | +++ [194] |  |  |  |  |  |  |
| O | *Strombidium kahli* |  |  |  | ++ |  |  |  |  | +++ [244] |  |
| O | *Strombidium kryale* | +++ [245] |  |  |  |  |  |  |  |  |  |
| O | *Strombidium lagenula* |  |  | ++ | +++ [209] |  | + |  | ++ |  | + |
| O | *Strombidium latum* |  | + | + | ++ |  |  |  |  | +++ [8] |  |
| O | *Strombidium lingulum* |  |  |  |  |  | +++ [19] |  |  |  |  |
| O | *Strombidium lynni* |  |  |  | ++ |  | +++ [217] |  |  |  |  |
| O | *Strombidium macronucleatum* |  |  |  | +++ [236] |  |  |  |  |  | + |
| O | *Strombidium maedai* |  |  |  | +++ [194] |  |  |  |  |  |  |
| O | *Strombidium meganucleatum* |  |  |  |  |  |  |  | ++ |  |  |
| O | *Strombidium minor* |  | + |  | +++ [246] |  | + |  |  |  | + |
| O | *Strombidium obliquum* |  |  |  | +++ [8] |  | + |  |  |  | + |
| O | *Strombidium oblongum* |  |  | ++ | +++ [196, 200] | ++ | + |  |  |  |  |
| O | *Strombidium oculatum* |  |  | +++ [232] | +++ [46] |  |  |  |  | + |  |
| O | *Strombidium opisthostomum* |  |  |  |  |  |  |  |  |  | +++ [216] |
| O | *Strombidium paracalkinsi* |  |  |  |  |  | +++ [247] |  |  |  |  |
| O | *Strombidium parastylifer* |  |  |  |  |  | +++ [193] |  |  |  |  |
| O | *Strombidium pollostomum* |  |  |  | +++ [194] |  |  |  |  |  |  |
| O | *Strombidium purpureum* |  |  |  | + |  |  |  |  | ++ |  |
| O | *Strombidium rapulum* |  |  |  |  |  | +++ [24, 248] |  |  |  |  |
| O | *Strombidium rassoulzadegani* |  |  |  | +++ [45] | ++ |  |  |  |  |  |
| O | *Strombidium rhynchum* |  |  | + | +++ [196] |  | + |  |  |  | + |
| O | *Strombidium sphaericum* |  |  | + | +++ [194] |  |  |  |  |  |  |
| O | *Strombidium stylifer* |  | + |  | ++ |  | +++ [222] |  | ++ | +++ [242] | + |
| O | *Strombidium sulcatum* | + | + | ++ | +++ [1, 249] | + | ++ | + | + | + | + |
| O | *Strombidium suzukii* |  |  |  |  |  | +++ [193] |  |  |  |  |
| O | *Strombidium syowaense* | +++ [250] |  |  |  |  |  |  |  |  |  |
| O | *Strombidium taylori* |  |  |  | ++ |  | +++ [217] |  |  |  |  |
| O | *Strombidium tintinnodes* |  | + | +++ [221] | ++ |  | +++ [213] |  |  |  |  |
| O | *Strombidium tressum* |  |  |  | +++ [196] |  | ++ |  | + | ++ | + |
| O | *Strombidium triquetrum* |  |  |  | +++ [156] |  |  |  |  |  |  |
| O | *Strombidium turcicum* |  |  | +++ [251] |  |  | + |  |  |  |  |
| O | *Strombidium ventropinnum* |  |  |  | + |  | +++ [217] |  |  |  | + |
| O | *Strombidium vestitum* |  |  | ++ | +++ [156, 200] |  | + |  |  | ++ | + |
| O | *Strombidium wulffi* | + | +++ [252] | ++ | +++ [196] |  | ++ |  |  |  | + |
| O | ***Tontonia*** | +++ | + | ++ | +++ |  | ++ | + |  | + | + |
| O | *Tontonia antarctica* | +++ [199] |  |  |  |  |  |  |  |  |  |
| O | *Tontonia appendiculariformis* |  | + | ++ | +++ [253] |  | ++ | + |  | + | + |
| O | ***Varistrombidium*** |  |  |  |  |  | +++ |  |  | +++ |  |
| O | *Varistrombidium kielum* |  |  |  |  |  | +++ [193] |  |  | +++ [8] |  |
| Total number of genera | | 11 | 14 | 15 | 18 | 7 | 23 | 5 | 10 | 14 | 12 |
| Total number of morphospecies | | 28 | 38 | 48 | 95 | 16 | 94 | 9 | 25 | 36 | 46 |
| Total number of endemic species | | 6 | 0 | 0 | 1 | 0 | 2 | 0 | 0 | 0 | 3 |
| Percentage of endemics [%] | | 25 | 0 | 0 | 1 | 0 | 2 | 0 | 0 | 0 | 7 |
| Total number of species descriptions /redescriptions | | 15 | 3 | 11 | 74 | 0 | 52 | 0 | 0 | 14 | 3 |

^1^ Antarctic Sea: area more than 60°S.

^2^ Arctic Sea: area more than 60°N; includes Greenland Sea, Norwegian Sea, Barents Sea, White Sea, Kara Sea, Laptev Sea, East Siberian Sea, Bering Sea, Chuckchee Sea, Beaufort Sea, Northwest Passages, Baffin Bay, Davis Strait, Hudson Bay, and Hudson Strait.

^3^ Mediterranean Sea: includes the Strait of Gibraltar, Alboran Sea, Iberian Sea, Ligurian Sea, Tyrrhenian Sea, Ionian Sea, Adriatic Sea, Sea of Marmara, Bosporus, and Aegean Sea.

^4^ North Atlantic: area between the equator and 60°N; includes North Sea, Irish Sea, English Channel, Bay of Biscay, Labrador Sea, Gulf of St. Lawrence, Bay of Fundy, Gulf of Mexico, and Caribbean Sea.

^5^ South Atlantic: area between the equator, 60°S, the meridian of Cape Horn (~ 67°W), and 20°E.

^6^ North Pacific: area between the equator and 60°N; includes the East Indian Archipelago, Gulf of Thailand, South China Sea, East China Sea, Yellow Sea, Japan Sea, Inland Sea, Sea of Okhotsk, Golf of Alaska, and Gulf of California.

^7^ South Pacific: area between the equator, 60°S, ~ 147°E, and ~ 67°W; includes Tasman Sea, Coral Sea, Solomon Sea, and Bismarck Sea.

^8^ Indian Ocean: area between 20°E and ~ 147°E and less than 60°S; includes the Gulf of Aqaba, Red Sea, Gulf of Aden, Arabian Gulf, Gulf of Oman, Laccadive Sea, Bay of Bengal, Andaman, Mozambique Channel, Malacca and Singapore Straits, Great Australian Bight, and Bass Strait.

^9^ Baltic Sea: includes the Gulf of Bothnia, Gulf of Finland, Gulf of Riga, Kattegat, Sounds, Belts, and Skagerrak.

^10^ Black Sea: includes Sea of Azov.

**References**

1. Claparède É, Lachmann J (1859) Études sur les infusoires et les rhizopodes. Mém Inst Natn Genev 6 (year 1858): 261–482 + Plates 14–24.

8. Kahl A (1932) Urtiere oder Protozoa I: Wimpertiere oder Ciliata (Infusoria) 3. Spirotricha. Tierwelt Dtl 25: 399–650.

19. Montagnes DJS, Humphrey E (1998) A description of occurrence and morphology of a new species of red-water forming *Strombidium* (Spirotrichea, Oligotrichia). J Eukaryot Microbiol 45: 502–506.

23. Xu D, Sun P, Song W, Warren A (2008) Studies on a new endocommensal ciliate, *Strombidium foissneri* nov. sp. (Ciliophora, Oligotrichida), from the intestine of the sea urchin *Hemicentrotus pulcherrimus* (Camarodonta, Echinoida). Denisia 23: 273–278.

24. Xu D, Song W, Sun P, Chen X (2006) Morphology and infraciliature of the oligotrich ciliate *Strombidium rapulum* (Yagiu, 1933) Kahl, 1934 (Protozoa, Ciliophora, Oligotrichida) from the intestine of sea urchin *Hemicentrotus pulcherrimus* Agassiz. Zootaxa 1113: 33–40.

37. Agatha S, Strüder-Kypke MC, Beran A, Lynn DH (2005) *Pelagostrobilidium neptuni* (Montagnes and Taylor, 1994) and *Strombidium biarmatum* nov. spec. (Ciliophora, Oligotrichea): phylogenetic position inferred from morphology, ontogenesis, and gene sequence data. Eur J Protistol 41: 65–83.

40. Kim Y-O, Suzuki T, Taniguchi A (2002) A new species in the genus *Cyrtostrombidium* (Ciliophora, Oligotrichia, Oligotrichida): its morphology, seasonal cycle and resting stage. J Eukaryot Microbiol 49: 338–343.

45. McManus GB, Xu D, Costas BA, Katz LA (2010) Genetic identities of cryptic species in the *Strombidium* *stylifer*/*apolatum*/*oculatum* cluster, including a description of *Strombidium rassoulzadegani* n. sp. J Eukaryot Microbiol 57: 369–378.

46. Montagnes DJS, Lowe CD, Poulton A, Jonsson PR (2002) Redescription of *Strombidium oculatum* Gruber 1884 (Ciliophora, Oligotrichia). J Eukaryot Microbiol 49: 329–337.

58. Kim JS, Jeong HJ, Strüeder-Kypke MC, Lynn DH, Kim S et al. (2005) *Parastrombidinopsis shimi* n. gen., n. sp. (Ciliophora: Choreotrichia) from the coastal waters of Korea: morphology and small subunit ribosomal DNA sequence. J Eukaryot Microbiol 52: 514–522.

60. Modeo L, Petroni G, Rosati G, Montagnes DJS (2003) A multidisciplinary approach to describe protists: redescriptions of *Novistrombidium testaceum* Anigstein 1914 and *Strombidium inclinatum* Montagnes, Taylor, and Lynn 1990 (Ciliophora, Oligotrichia). J Eukaryot Microbiol 50: 175–189.

64. Tsai S-F, Xu D, Chung C-C, Chiang K-P (2008) *Parastrombidinopsis minima* n. sp. (Ciliophora: Oligotrichia) from the coastal waters of northeastern Taiwan: morphology and small subunit ribosomal DNA sequence. J Eukaryot Microbiol 55: 567–573.

141. Jeong HJ, Kim JS, Kim S, Song JY, Lee I et al. (2004) *Strombidinopsis jeokjo* n. sp. (Ciliophora: Choreotrichida) from the coastal waters off western Korea: morphology and small subunit ribosomal DNA gene sequence. J Eukaryot Microbiol 51: 451–455.

156. Agatha S, Riedel-Lorjé JC (1997) Morphology, infraciliature, and ecology of halteriids and strombidiids (Ciliophora, Oligotrichea) from coastal brackish water basins. Arch Protistenk 148: 445–459.

192. Lynn DH, Montagnes DJS (1988) Taxonomic descriptions of some conspicuous species of strobilidiine ciliates (Ciliophora: Choreotrichida) from the Isles of Shoals, Gulf of Maine. J Mar Biol Ass UK 68: 639–658.

193. Xu D, Warren A, Song W (2009) Oligotrichs. In: Song W, Warren A, Hu X, editors. Free-living Ciliates in the Bohai and Yellow Seas, China. Beijing: Science Press. pp. 307–351.

194. Lynn DH, Gilron GL (1993) Strombidiid ciliates from coastal waters near Kingston Harbour, Jamaica (Ciliophora, Oligotrichia, Strombidiidae). J Mar Biol Ass UK 73: 47–65.

195. Meunier A (1910) Campagne arctique de 1907. Microplankton des mers de Barents et de Kara. Bruxelles: Bulens. i–xviii + 355 p. + Plates 1–36.

196. Lynn DH, Montagnes DJS, Small EB (1988) Taxonomic descriptions of some conspicuous species in the family Strombidiidae (Ciliophora: Oligotrichida) from the Isles of Shoals, Gulf of Maine. J Mar Biol Ass UK 68: 259–276.

197. Montagnes DJS, Lynn DH, Stoecker DK, Small EB (1988) Taxonomic descriptions of one new species and redescription of four species in the family Strombidiidae (Ciliophora, Oligotrichida). J Protozool 35: 189–197.

198. Lohmann H (1908) Untersuchungen zur Feststellung des vollständigen Gehaltes des Meeres an Plankton. Wiss Meeresunters, Abt Kiel 10: 129–370 + Plates 9–17.

199. Petz W, Song W, Wilbert N (1995) Taxonomy and Ecology of the Ciliate Fauna (Protozoa, Ciliophora) in the Endopagial and Pelagial of the Weddell Sea, Antarctica. Stapfia 40: 223 p.

200. Leegaard C (1915) Untersuchungen über einige Planktonciliaten des Meeres. Nytt Mag Naturvid 53: 1–37.

201. Liu W, Yi Z, Lin X, Al-Rasheid KAS (2011) Morphologic and molecular data suggest that *Lynnella semiglobulosa* n. g., n. sp. represents a new family within the subclass Choreotrichia (Ciliophora, Spirotrichea). J Eukaryot Microbiol 58: 43–49.

202. Liu W, Xu D, Lin X, Li J, Gong J et al. (2009) *Novistrombidium sinicum* n. sp. and *Novistrombidium orientale* n. sp. (Protozoa: Ciliophora): two new oligotrich ciliates from a mangrove wetland, South China. J Eukaryot Microbiol 56: 459–465.

203. Anigstein L (1913) Über *Strombidium testaceum* nov. spec. eine marine oligotriche Ciliate. Arch Protistenk 32: 79–110 + Plates 1, 2.

204. Song W, Wang M, Warren A (2000) Redescriptions of three marine ciliates, *Strombidium elegans* Florentin, 1901, *Strombidium sulcatum* Claparède & Lachmann, 1859 and *Heterostrombidium paracalkinsi* Lei, Xu & Song, 1999 (Ciliophora, Oligotrichida). Eur J Protistol 36: 327–342.

205. Xu D, Song W, Warren A (2006) Morphology and infraciliature of two new species of marine oligotrich ciliates (Ciliophora: Oligotrichida) from China. J Nat Hist (London) 40: 1287–1299.

206. Song W (2005) Taxonomic description of two new marine oligotrichous ciliates (Protozoa, Ciliophora). J Nat Hist (London) 39: 241–252.

207. Corliss JO, Snyder RA (1986) A preliminary description of several new ciliates from the Antarctica, including *Cohnilembus grassei* n. sp. Protistologica 22: 39–46.

208. Montagnes DJS, Taylor FJR (1994) The salient features of five marine ciliates in the class Spirotrichea (Oligotrichia), with notes on their culturing and behaviour. J Eukaryot Microbiol 41: 569–586.

209. Fauré-Fremiet E (1924) Contribution a la connaissance des infusoires planktoniques. Bull Biol Fr Belg Suppl 6: 1–171.

210. Xu D, Song W, Warren A, Roberts D, Hu X (2007) Redescriptions of two marine planktonic ciliates from China, *Parastrombidium faurei* (Kahl, 1932) Maeda, 1986 and *Strombidium capitatum* (Leegaard, 1915) Kahl, 1932 (Ciliophora, Oligotrichea). Eur J Protistol 43: 27–35.

211. Alekperov IK, Buskey E, Snegovaya N (2008) The free-living ciliates of the Mexican Gulf coast near Port Aransas city and its suburbs (South Texas, USA). Protistology 5 (year 2007/2008): 101–130.

212. Montagnes DJS, Lynn DH (1988) A new species of *Tontonia* (Ciliophora: Oligotrichida) from the Isles of Shoals, Maine, U.S.A. Trans Am Microsc Soc 107: 305–308.

213. Song W, Bradbury PC (1998) Studies on some new and rare reported marine planktonic ciliates (Ciliophora: Oligotrichia) from coastal waters in north China. J Mar Biol Ass UK 78: 767–794.

214. Suzuki T, Song W (2001) A redescription of *Tontonia cornuta* (Leegaard, 1915) comb. nov., a planktonic oligotrichous ciliate (Ciliophora: Oligotrichia) from the northern Pacific Ocean. Hydrobiologia 457: 119–123.

215. Agatha S, Riedel-Lorjé JC (1998) Morphology, infraciliature, and ecology of some strobilidiine ciliates (Ciliophora, Oligotrichea) from coastal brackish water basins of Germany. Eur J Protistol 34: 10–17.

216. Tucolesco J (1962) Études protozoologiques sur les eaux roumaines. I. Espèces nouvelles d’infusoires de la mer Noire et des bassins salés paramarins. Arch Protistenk 106: 1–36.

217. Martin AJ, Montagnes DJS (1993) Winter ciliates in a British Columbian fjord: six new species and an analysis of ciliate putative prey. J Eukaryot Microbiol 40: 535–549.

218. Xu D, Song W, Lin X, Warren A (2006) On two marine oligotrich ciliates, *Spirostrombidium agathae* n. sp. and *S. schizostomum* (Kahl, 1932) n. comb. from China, with a key to the identification of seven well-characterized *Spirostrombidium* spp. (Ciliophora: Oligotrichida). Acta Protozool 45: 433–442.

219. Xu D, Song W (2006) Hapantotypification and morphological redescription of the marine planktonic ciliate, *Spirostrombidium cinctum* (Kahl, 1932) Petz, Song et Wilbert, 1995 (Ciliophora: Oligotrichida). Acta Protozool 45: 17–25.

220. Song W, Wilbert N, Warren A (1999) Three new entocommensal ciliates from digestive tract of sea urchins of the Weddell Sea, Antarctica (Protozoa, Ciliophora). Polar Biol 22: 232–240.

221. Entz G, Sr (1884) Über Infusorien des Golfes von Neapel. Mitt Zool Stn Neapel 5: 289–444 + Plates 20–25.

222. Song W, Packroff G (1997) Taxonomische Untersuchungen an marinen Ciliaten aus China mit Beschreibungen von zwei neuen Arten, *Strombidium globosaneum* nov. spec. und *S. platum* nov. spec. (Protozoa, Ciliophora). Arch Protistenk 147 (year 1996/1997): 331–360.

223. Wang CC (1934) Notes on the marine infusoria of Amoy. Rep Mar Biol Ass China 3: 50–70.

224. Sauerbrey E (1928) Beobachtungen über einige neue oder wenig bekannte marine Ciliaten. Arch Protistenk 62: 355–407 + Plates 22–26.

225. Lei Y, Xu K, Song W (1999) Free living ciliates from marine farming ponds. In: Song W, editor. Progress in Protozoology. Qingdao: Qingdao Ocean Univ. Press. pp. 269–295 (in Chinese).

226. Stein F (1867) Der Organismus der Infusionsthiere nach eigenen Forschungen in systematischer Reihenfolge bearbeitet. II. Abtheilung. 1) Darstellung der neuesten Forschungsergebnisse über Bau, Fortpflanzung und Entwickelung der Infusionsthiere. 2) Naturgeschichte der heterotrichen Infusorien. Leipzig: Engelmann W. i–viii + 355 p. + Plates 1–16.

227. Suzuki T, Han M-S (2000) A study on a new species of *Tontonia* (Ciliophora: Oligotrichida) from the East China Sea and adjacent sea areas. J Mar Biol Ass UK 80: 989–994.

228. Tsai S-F, Chen J-Y, Chiang K-P (2010) *Spirotontonia taiwanica* n. sp. (Ciliophora: Oligotrichida) from the coastal waters of northeastern Taiwan: morphology and nuclear small subunit rDNA sequence. J Eukaryot Microbiol 57: 429–434.

229. Lynn DH, Montagnes DJS, Dale T, Gilron GL, Strom SL (1991) A reassessment of the genus *Strombidinopsis* (Ciliophora, Choreotrichida) with descriptions of four new planktonic species and remarks on its taxonomy and phylogeny. J Mar Biol Ass UK 71: 597–612.

230. Xu R, Bai Q (1998) *Strombidinopsis grandis*: a new species of *Strombidinopsis*. Tropic Oceanology 17: 40–43 (in Chinese with English summary).

231. Fauré-Fremiet E (1910) Sur deux infusoires planktoniques. Bull Soc Zool Fr 35: 226–227.

232. Gruber A (1884) Die Protozoen des Hafens von Genua. Nova Acta Acad Caesar Leop Carol 46: 473–539 + Plates 7–11.

233. Agatha S (2003) Redescription of *Strombidinopsis minima* (Gruber, 1884) Lynn et al., 1991 (Protozoa, Ciliophora), with notes on its ontogenesis and distribution. Eur J Protistol 39:­ 233–244.

234. Busch W (1930) Ueber marine *Strombidium*-Arten aus der antarktischen allgemeinen Ostströmung. Abh Mus Nat Heimatk Magdeb 6: 83–87.

235. Wilbert N, Song W (2005) New contributions to the marine benthic ciliates from the Antarctic area, including description of seven new species (Protozoa, Ciliophora). J Nat Hist (London) 39: 935–973.

236. Dragesco J (1960) Ciliés mésopsammiques littoraux. Systématique, morphologie, écologie. Trav Stn Biol Roscoff (NS) 12: 1–356.

237. Agatha S (2003) Morphology and ontogenesis of *Novistrombidium apsheronicum* nov. comb. and *Strombidium arenicola* (Protozoa, Ciliophora): a comparative light microscopical and SEM study. Eur J Protistol 39: 245–266.

238. Fauré-Fremiet E (1932) *Strombidium calkinsi*, a new thigmotactic species. Biol Bull Mar Biol Lab, Woods Hole 62: 201–204.

239. Buddenbrock W von (1922) Über eine neue *Strombidium*-Art aus Helgoland (*Str. clavellinae*). Arch Protistenk 45: 129–132.

240. Wailes GH (1929) Marine ciliates of the genus *Laboea* from British Columbia with description of a new species. Annls Protist 2: 125–126.

241. Gourret P, Roeser P (1888) Contribution à l’étude des protozoaires de la Corse. Archs Biol 8: 139–204 + Plates 13–15.

242. Levander KM (1894) Materialien zur Kenntniss der Wasserfauna in der Umgebung von Helsingfors, mit besonderer Berücksichtigung der Meeresfauna. I. Protozoa. Acta Soc Fauna Flora Fenn 12: 1–115 + Plates 1–3.

243. Montagnes DJS, Taylor FJR, Lynn DH (1990) *Strombidium inclinatum* n. sp. and a reassessment of *Strombidium sulcatum* Claparède and Lachmann (Ciliophora). J Protozool 37: 318–323.

244. Bock KJ (1952) Über einige holo- und spirotriche Ciliaten aus den marinen Sandgebieten der Kieler Bucht. Zool Anz 149: 107–115.

245. Petz W (1994) Morphology and morphogenesis of *Strombidium kryalis* nov. spec. (Ciliophora, Strombidiida) from Antarctic sea ice. Arch Protistenk 144: 185–195.

246. Calkins GN (1902) Marine protozoa from Woods Hole. Bull US Fish Commn 21 (year 1901): 413–468.

247. Lei Y, Xu K, Song W (1999) Morphological studies on a new ciliate, *Heterostrombidium paracalkinsi* nov. spec. from mariculture water (Ciliophora, Oligotrichida). Chin J Appl Environ Biol 5: 181–184 (in Chinese with English summary and explanations of figures and tables).

248. Yagiu R (1933) Studies on the ciliates from the intestine of *Anthocidaris crassispina* (A. Agassiz). J Sci Hiroshima Univ, Ser B, Div 1, Vol 2: 211–222 + Plates 1, 2.

249. Granda AP, Montagnes DJS (2003) An improved description of *Strombidium sulcatum* Claparède & Lachmann, 1859 (Ciliophora) from slides of Fauré-Fremiet, and a designation of type material. J Eukaryot Microbiol 50: 422–426.

250. Hada Y (1970) The protozoan plankton of the Antarctic and Subantarctic Seas. JARE Sci Rep, Ser E 31: 1–51.

251. Hovasse R (1932) Trois infusoires planktoniques du Bosphore. Archs Zool Exp Gén 73 (Notes et Revue): 1–8.

252. Wulff A (1919) Ueber das Kleinplankton der Barentssee. Wiss Meeresunters, Abt Helgoland 13: 95–125 + Plates 1–4.

253. Fauré-Fremiet E (1914) Deux infusoires planctoniques *Tontonia appendiculariformis* (n. gen., n. sp.) et *Climacostomum diedrum* (n. sp.). Arch Protistenk 34: 95–107.
